# Viburnum Prunifolium Unreliable in Abortion

**Published:** 1882-03-20

**Authors:** A. G. Smythe

**Affiliations:** Mississippi


### Southern Medical Record:

EDITORS:

T. S. Powell, M.D. W. T. Goldsmith, M.D. R. C. Word, M.D.

*R. C. WORD, M.D., Managing Editor.*

☞All Communications and Letters on Business connected with the Record must be addressed to the Managing Editor.

Vol. XII. ATLANTA, GA., MARCH 20, 1882. No. 3.

### ORIGINAL AND SELECTED ARTICLES.

### VIBURNUM PRUNIFOLIUM UNRELIABLE IN ABORTION—A REJOINDER.

BY A. G. SMYTHE, M. D., OF MISSISSIPPI.

Gentlemen—I ask space for some remarks in reply to Dr. Thomas F. Houston's article in your last issue. The Doctor's very confident expressions as to the virtues of viburnum prunifolium, though doubtless honestly made on his part, do not convince me of any error in my views, or set aside the facts and experiments upon which they were based. And as to doctors disagreeing, that is a thing of daily occurrence in all the departments of science.

Medicine is not an exact science, and cannot be demonstrated by a mathematical elucidation, and until a better method of arriving at correct conclusions than any now known to the medical men, there will be continual differences of opinion and conclusions, in all of its departments.

In my article in the December number of the Record, there was no wish or intention to invite a discussion upon the merits of the remedy in question. (There is nothing to discuss.) The desire was simply to warn the confiding members of the profession to avoid the rocks, shoals and quicksands upon which I have so often been wrecked. *Badinage aside.* The virtues of the remedy were sounded through the literature of the day, and so far as I am informed, without question or contradiction, and from my unfortunate experience, I could not permit it to pass any longer without giving the professional public my observations. It was in no prurient desire to appear in public; but after repeated postponings that it was done. The first suggestion was to ask its appearance in the columns of the *Therapeutic Gazette.* But upon reflection concluded that as that journal was principally devoted to the advocacy of new remedies and new preparations, that it might not appear to be compatible with its objects and interests. It was then suggested to offer it to one of my favorites, THE SOUTHERN MEDICAL RECORD. I am happy to note the spirit of generous fairness manifested by the *Therapeutic Gazette* in copying the article in the February number.

I regret exceedingly that it was my misfortune to meet with such a signal disappointment in the trial of the remedy, if remedy it is. In the first place, to fail to treat that or any other case successfully. In the second place, to fail to find a new and reliable remedy. In the third place, to be forced to differ with my personal and professional friends and brethren; all of which is very unpleasant and unprofitable, to say the least.

I am charged with not having given it a FAIR TRIAL. Ten pages of notes of cases could be given. When making trials and testing remedies, I do not come to hasty and inconsiderate conclusions. Various forms and doses were given to different patients, at different times. In the outset of trials of the remedy, I had several subjects who were habitual abortionists, and flattered myself that I would now have no more cause to mourn over failure and disappointments. In a general way I am not sanguine of success; but for once I was carried away with the hope that now I had the remedy of remedies in this, to me, troublesome derangement. Trial after trial (FAIR TRIALS) upon the same and upon different patients were made. And I reiterate it, that in no case when it was relied upon, did it succeed. Some of the cases became dissatisfied and discharged me, others continued. I abandoned its use, as before said, in disappointment, mortification and disgrace. The latter of which I have not recovered from yet. I have adopted a different treatment with different remedies with as good success as could be hoped for in any treatment now known. Having overcome the disposition to miscarry in all the cases who continued under my treatment, all having had more or less children, and have no chronic or habitual cases of abortion or miscarriage at present.

I may be mistaken, but yet think that much, if not all the credit attributed to this much vaunted and highly extolled remedy, is due to the adjuvants and other means used in the treatment of threatened abortions. All of us are, more or less, anxious to give relief as soon as possible; and are, more than likely, in urgent cases, to give more than one remedy, unless the one given relieves the trouble immediately, and more especially is it the case if the remedy is new or uncertain in its action. I am free to admit that such is the habit with myself, unless it is when making a direct experiment, as was the case in the trials I made with viburnum prunifolium. In no case do I recommend the use of any remedy—either new or old—in any new or special way, without giving it numerous, fair and independent trials. True, I may report its use in a single or in a few cases. May and have suggested remedies which I never used, or even ever heard of being used. But in no case urge and recommend the use of an article for the reason that in one or a few cases it appears to do well. There are a number of articles in our Materia Medica that I think are good remedies, which will not do to rely upon implicitly.

In a few cases of chronic diarrhœa I have had the most happy effects from the use of the fluid extract of guarana, yet in other cases it has failed. An untold number of similar instances might be cited.

In replying to the article of Dr. Houston, there was no argument or reason to give for the dogmatic assertion that viburnum prunifolium had failed as a remedy in threatened abortion—a mere relation of what had, and what had not taken place. I have now given the only reason for making it public; and trust that the profession, and especially the readers of this Journal, will pardon the first article, and accept this in explanation, and as an apology for occupying the space in the RECORD, and their time in reading the same.

